# Pulmonary vascular volume is associated with DLCO and fibrotic score in idiopathic pulmonary fibrosis: an observational study

**DOI:** 10.1186/s12880-022-00803-8

**Published:** 2022-04-23

**Authors:** Wen-Jui Wu, Wei-Ming Huang, Chia-Hao Liang, Chun-Ho Yun

**Affiliations:** 1 Division of Pulmonary and Critical Care Medicine, Mackay Memorial Hospital, Taipei City, Taiwan; 2 Department of Radiology, Mackay Memorial Hospital, Taipei City, Taiwan; 3 Department of Medicine, Mackay Medical College, New Taipei City, Taiwan; 4 Mackay Junior College of Medicine, Nursing, and Management, New Taipei City, Taiwan; 5 Department of Biomedical Imaging and Radiological Sciences, National Yang Ming Chiao Tung University, Taipei, Taiwan; 6 Department of Radiology, School of Medicine, College of Medicine, Taipei Medical University, Taipei, Taiwan; 7 Department of Radiology, Wan Fang Hospital, Taipei Medical University, Taipei, Taiwan

**Keywords:** Idiopathic pulmonary fibrosis, Pulmonary vascular volume, Fibrotic score, DLCO

## Abstract

**Background:**

Idiopathic pulmonary fibrosis (IPF) is a disease that primarily occurs in elderly individuals. However, it is difficult to diagnose and has a complex disease course. High-resolution computed tomography (HRCT) and lung function testing are crucial for its diagnosis and follow-up. However, the correlation of HRCT findings with lung function test results has not been extensively investigated.

**Methods:**

This study retrospectively analysed the medical records and images of patients with IPF. Patients with evident emphysema and lung cancer were excluded. The diagnosis of all the included cases was confirmed following a discussion among specialists from multiple disciplines. The correlation of HRCT findings, including fibrotic score, HRCT lung volume, pulmonary artery trunk (PA) diameter and pulmonary vascular volume (PVV), with lung function test parameters, such as forced vital capacity (FVC) and diffusing capacity for carbon monoxide (DLCO), was analysed.

**Results:**

A total of 32 patients were included. Higher fibrotic and PVV scores were significantly correlated with lower DLCO (r =  − 0.59, *p* = 0.01; r =  − 0.43, *p* = 0.03, respectively) but not with FVC. Higher PVV score significantly correlated with higher fibrotic score (r = 0.59, *p* < 0.01) and PA diameter (r = 0.47, *p* = 0.006).

**Conclusion:**

Our study demonstrated the structural and functional correlation of IPF. The extent of lung fibrosis (fibrotic score) and PVV score were associated with DLCO but not with FVC. The PA diameter, which reflects the pulmonary artery pressure, was found to be associated with the PVV score.

## Background

Idiopathic pulmonary fibrosis (IPF) is a chronic, progressive, fibrosing interstitial pneumonia of unknown cause that primarily affects elderly individuals and has an extremely poor prognosis, with a median survival of 3–5 years after diagnosis [1]. The diagnosis of IPF is difficult and usually warrants the need for a multidisciplinary discussion (MDD) with a pulmonologist, radiologist and pathologist to make an accurate diagnosis [1]. Moreover, the disease course is complex and difficult to predict [2].

A lung function test is crucial to detect, diagnose and monitor the progression of IPF. Forcedvital capacity (FVC) and diffusing capacity for carbon monoxide (DLCO) levels are considered as the most valuable parameters of lung function tests for the diagnosis of IPF [3]. The rate of FVC decline has been used as a marker for disease progression because of its association with mortality [4], and DLCO measures another physiological deficiency (gas diffusion) associated with lung fibrosis. DLCO was reportedly also associated with pulmonary hypertension, an important comorbidity of IPF. The GAP index that combines age, sex and FVC and DLCO findings can predict mortality. However, the abnormalities of lung function tests on IPF may be diversed. FVC and DLCO may not be equally affected. Lung function test results may be normal in some patients with clinical or pathological IPF [3].

High-resolution computed tomography (HRCT) of the chest is another key examination for IPF. The fibrotic patterns on HRCT are important clues to classify and diagnose IPF [1]. Recently, tools were developed to quantify the chest HRCT findings for the diagnosis of IPF; the findings may include the extent of pulmonary fibrosis and volume of the pulmonary vasculature. Despite technological advances, the association of HRCT findings with lung function tests in terms of the diagnosis of IPF is still not extensively investigated. Moreover, there is still no consensus on the relationship between CT findings, pulmonary vasculature, the extent of fibrosis and lung function. The limited number of studies on this topic did not report unanimous results [5–8]. Therefore, this study aimed to demonstrate the relationship between the extent of fibrosis, pulmonary vasculature and lung function of patients with IPF.

## Methods

### Patient selection

This retrospective study on human subjects was approved by the local ethics committee (The Institutional Review Board of MacKay Memorial Hospital, Taipei Branch, Taipei, Taiwan). From December 2019 to April 2021, 106 cases discussed during the MDD were enrolled for further investigation. The MDD was conducted in accordance with the guidelines laid down for the treatment of IPF [9]. The MDD committee comprised pulmonologists, rheumatologists, radiologists and pathologists. The discussion and the diagnosis were based on the diagnostic algorithm proposed in the guideline. Patients with a confirmed IPF diagnosis were included, whereas those who were (1) diagnosed with non-IPF diseases (including connective tissue disease-related interstitial lung disease [CTD-ILD], lymphangioleiomyomatosis [LAM], lymphocytic interstitial pneumonia [LIP], chronic hypersensitivity pneumonitis [CHP], sarcoidosis, infection/airway disease, etc.); (2) diagnosed with an indeterminate usual interstitial pneumonia (UIP) pattern; (3) having concurrent pulmonary malignancy (primary or secondary); (4) without pulmonary function test results within 3 months of the CT scan and (5) having acute disease exacerbation (according to clinical conditions and CT images) as well as (6) those who could not be diagnosed with IPF at the MDD were excluded from the study. This study was performed in compliance with the Declaration of Helsinki, and the study protocol was approved by the Institutional Review Board at Mackay Memorial Hospital, Taipei, Taiwan (no. 21MMHIS180e). Data were anonymously analysed.

CT scans revealing emphysema that was determined to more than mild based on the Fleischner Society classification system [10, 11] were also excluded because the pulmonary vasculature would be affected by the emphysema [9].

### Clinical information

Patients’ characteristics (including age and gender) and pulmonary function tests (FVC, FVC%, DLCO and DLCO%) were recorded.

### CT imaging protocols

All CT scans were performed using a 128-slice (Somatom Definition AS, Siemens Healthcare, Forchheim, Germany) or 256-slice (Somatom Definition Flash, Siemens Healthcare, Forchheim, Germany) multidetector computed tomography (MDCT) scanner. CT images for all patients were identical with the same imaging parameters: a collimation of 128 × 0.6 or 256 × 0.6 mm, tube voltage of 120 kVp, tube current modulation, the gantry rotation speed of 0.5 s/r and reconstructed slice thickness of 1.5 mm in one whole breath-hold. The scan coverage was from the lung apex to the lowest hemidiaphragm. All images were acquired in a supine position and at full inspiration.

### Image analysis and lung function quantification

**Fig. 1:**
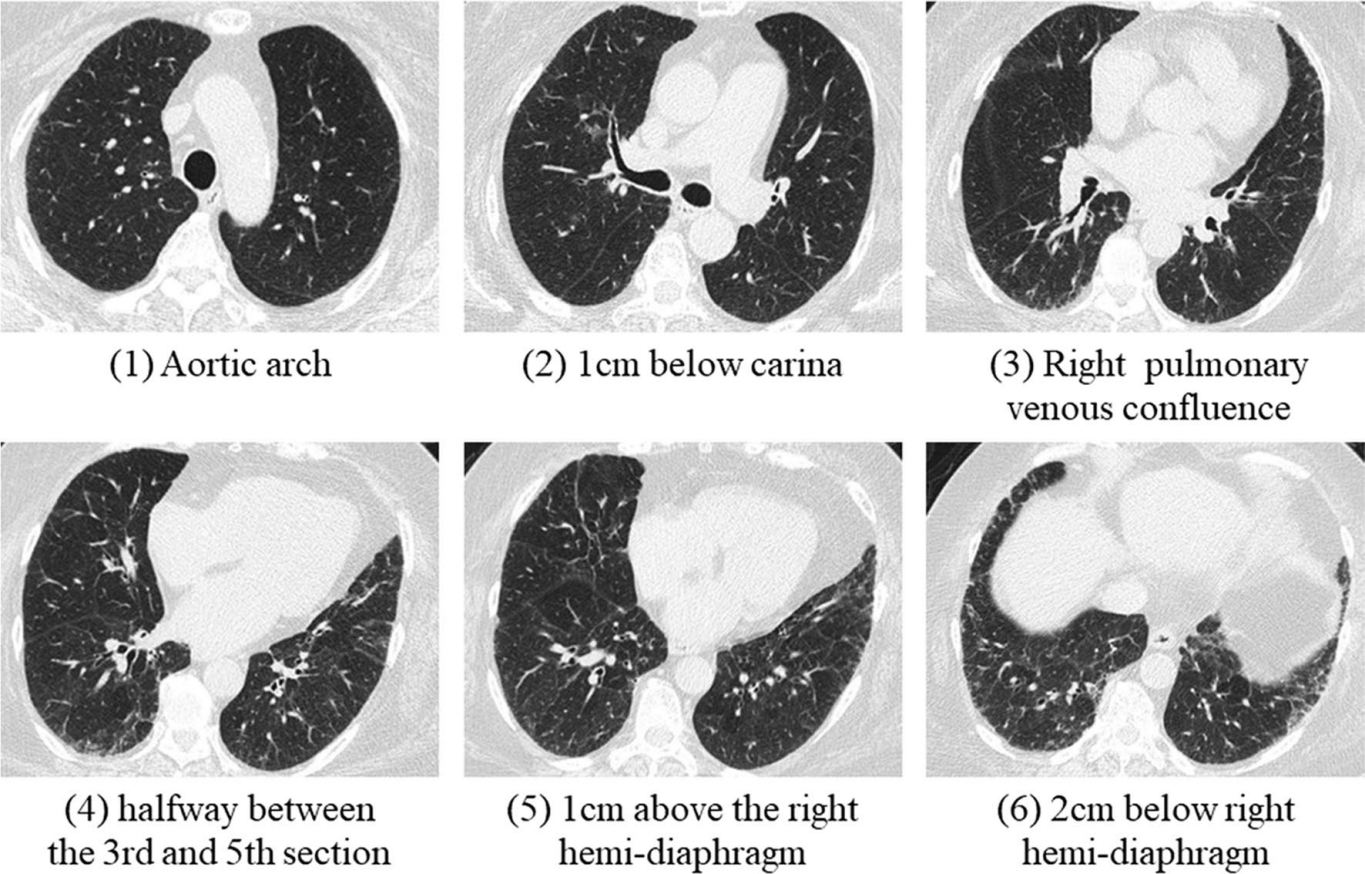
A 69-year-old woman was diagnosed with IPF with a probable UIP pattern as determined in the MDD. The percentage of fibrosis was calculated at each of these six levels, and the fibrotic score was the average percentage of the findings at these six sections

All the CT images were reviewed by two radiologists (W.H. and C.Y.) with 8 and 18 years of experience in examining chest CT images, respectively, who were blinded to information regarding the patients’ lung function. The fibrotic score ascertained made at six levels: 1) aortic arch, 2) 1 cm below the carina, 3) right pulmonary venous confluence, 4) halfway between the third and fifth section, 5) 1 cm above the right hemidiaphragm and 6) 2 cm below the right hemidiaphragm (Fig. 1) [12]. The proportion of the content with at least one of the following characteristics was scored to the nearest 5%: honeycombing, traction bronchiectasis, subpleural reticulation and ground-glass opacity with traction bronchiectasis in each section, and the fibrotic score was the average of the percentage in these six sections [13]. The short-axis diameter of the pulmonary artery (PA) trunk on axial sections of the mediastinal window at the PA bifurcation level was measured [14].

### The principles of quantification for the lung, emphysema and vessel volumes

**Fig. 2:**
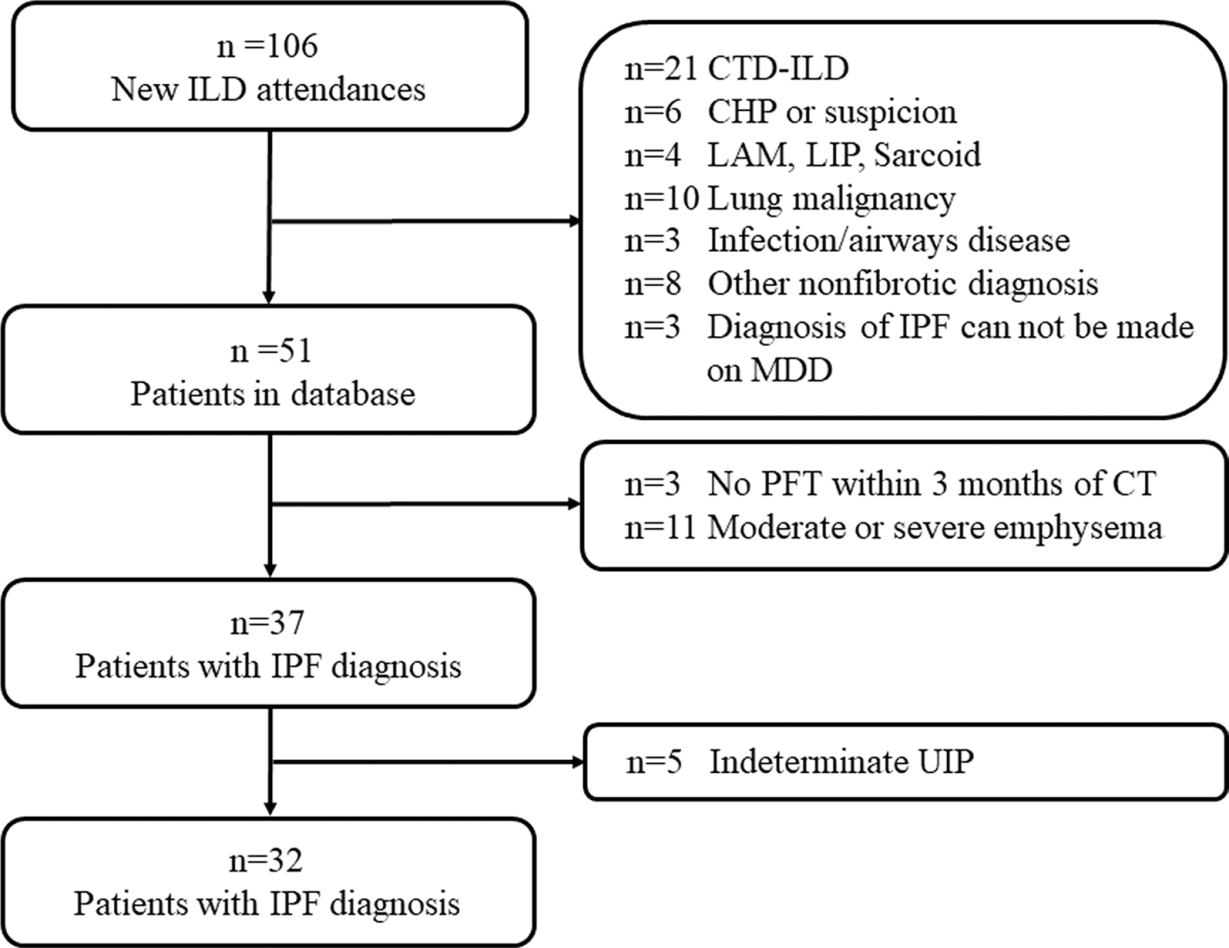
Flow chart depicting case selection of the retrospective study

We adopted commercial software (QUIBIM Precision 2.8, QUIBIM SL, Valencia, Spain) for lung segmentation, vessel extraction and emphysema extraction based on several steps of image thresholding and classification. The first transformation in the raw data domain is a preliminary segmentation of lung parenchyma using − 450 HU as the threshold value. The lung classification step involves the use of distance and the watershed transforms to localize the plane that passes between both lungs for accurate lung separation (Fig. 2).

**Fig. 3:**
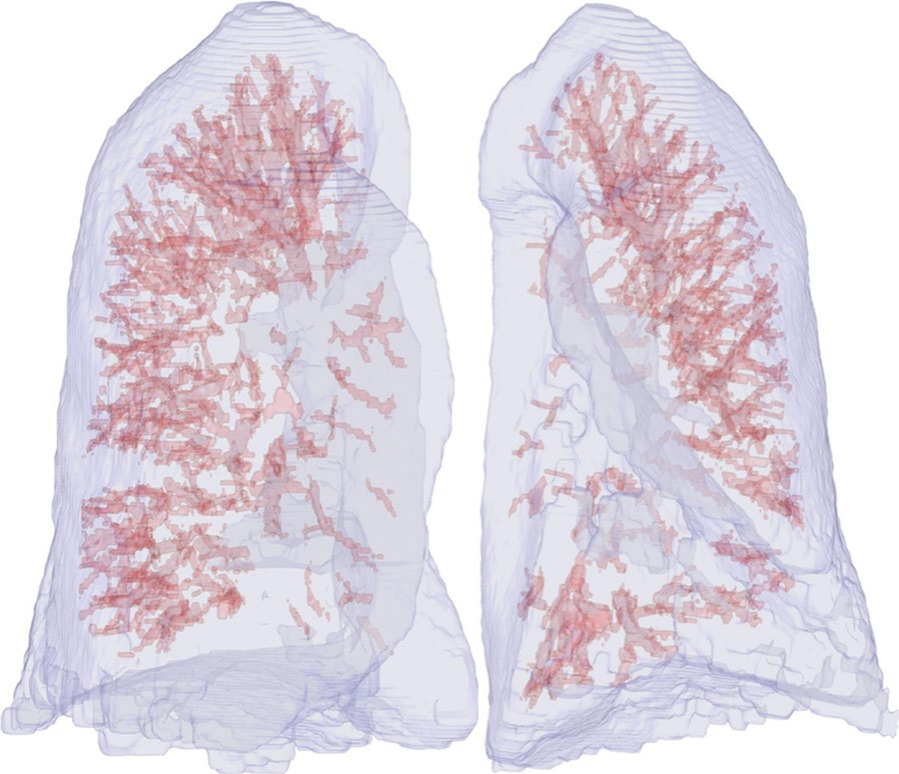
The 3D reconstruction and quantitative assessment of the pulmonary vessels and lung volumes of a 59-year-old man diagnosed with IPF (pulmonary vascular volume, 203.21 ml; lung volume, 4834.92 ml; and PVV score, 0.04)

The vessel-volume extraction algorithm by QUIBIM Precision was adopted by the technology Frangi filter, which uses the eigenvalue decomposition and vectors of the local Hessian matrix of CT to discriminate between plane-, blob- and tubular-like structures of the lung vessels (Fig. 3). As lung vessels have different radii in the chest region, it is significantly important to accurately extract vessel structures in a multi-scale framework using the algorithms of the Hessian matrix and the vessel measurements developed by Frangi [15]. The accuracy of vessel-volume evaluation and calculation has been well validated by Frangi’s publication [15], which reports an excellent area under the curve of 0.978, specificity of 0.900 and sensitivity of 0.973 at an optimal threshold higher than 0.9 in almost all cases.

The pulmonary vascular volume (PVV) score was calculated based on the ratio of the vessel and total lung volume.

The methodology for lung emphysema quantification included segmentation and extraction using a fixed threshold of − 950 HU. Based on this methodology, all pixel intensity values below the fixed threshold were considered as emphysema, whereas pixel values above the threshold were considered lung parenchyma. Relative volumes in the percentage were computed as the ratio between the total absolute volume occupied by the structure divided by the total lung volume.

### Statistical analysis

The correlation between lung function and CT parameters was examined using Spearman’s correlation. The lung function parameters, DLCO values and FVC values, were expressed as percentiles based on normal predicted values. The inter-rater reliability of the fibrotic score was assessed using the intraclass correlation coefficient (ICC). All tests were two-sided, and *p*-values of < 0.05 were considered statistically significant. The statistics were performed with R version 4.0.2 (R Foundation for Statistical Computing, Vienna, Austria).

## Results

A total of 106 cases were included in the MDD from December 2019 to April 2021. Among them, 55 who were diagnosed with conditions other than IPF were excluded. Among the remaining 51 patients, 3 were excluded as they did not perform a lung function test within 3 months of chest HRCT, 11 due to moderate to severe emphysema, and 5 due to subtle lung fibrosis and lack of confidence in their diagnosis of IPF. Finally, 32 patients diagnosed with IPF in the MDD were selected for undergoing investigation (Fig. 2).

### Basic characteristics

The basic characteristics of our population are presented in Table 1. Men accounted for 68.9% of the population, with a mean age of 74.7 (± 7.8) years, mean FVC (%) of 84.5% (± 22.5%) and mean DLCO of 63.0% (± 24.2%). CT parameters, including CT lung volume, PVVscore, fibrotic score and PA trunk diameter are also summarised in Table 1, which were 2959.3 (± 1047.1) cm3, 7.83 (± 3.01)%, 23.7 (± 10.9)% and 3.04 (± 0.46) cm, respectively.

**Table 1:** Background characteristics

	Patients (n = 32)
Age	74.7 (7.8)
Gender (M)	22 (68.9%)
FVC (%)	84.5 (22.5)
FEV1 (%)	88.8 (23.6)
DLCO (%)	63 (24.2)
TLC (%)	74 (16.4)
FS (%)	23.7 (10.9)
PVV score	7.83 (3.01)
CT-LV	2959.3 (1047.1)
PA	3.04 (0.46)

*FS* fibrotic score, *PVV* pulmonary vessel volume, *CT-LV* CT measured lung volume, *PA* pulmonary artery trunk diameter

### Reproducibility of the fibrotic score

**Fig. 4:**
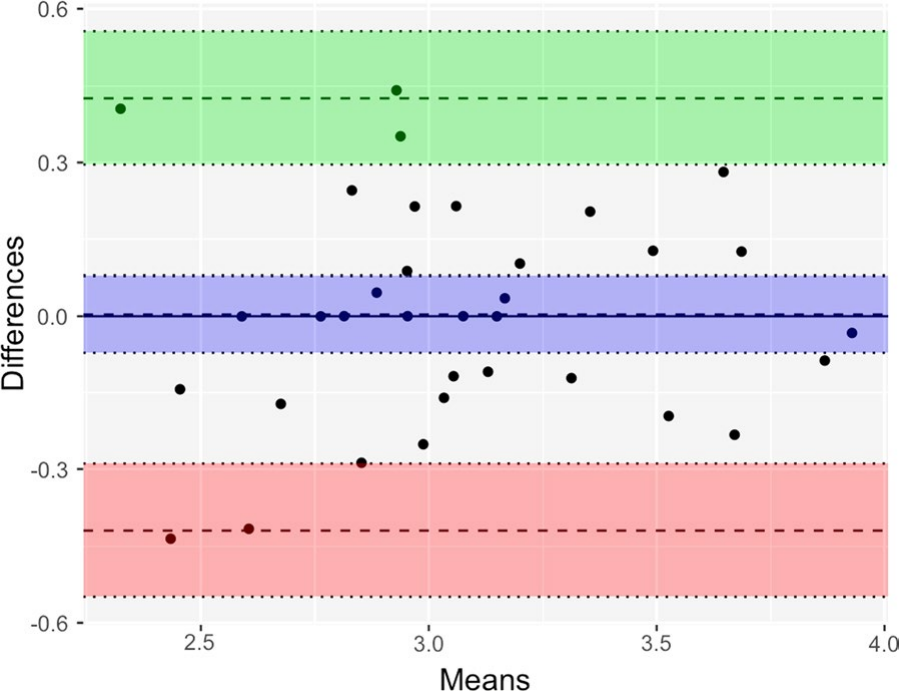
Bland–Altman plot for comparison of fibrotic scores evaluated by 2 radiologists. The fibrotic scores were log transformed. Mean bias = 0.0035 (95%CI: − 0.072, 0.079), upper LOA = 0.43(95%CI: 0.30, 0.56) and lower LOA =  − 0.42 (95%CI: − 0.29, − 0.55). The high reproducibility without consistent bias was demonstrated

Fibrotic scores estimated through the chest CT were used to evaluate inter-rater reliability. A total of 32 chest CTs were evaluated by two radiologists. The intraclass correlation coefficient (ICC) and Bland–Altman plot were used to evaluate the reproducibility of the fibrotic scores. As the scores were not normally distributed, log transformation was performed. The Bland–Altman plot is illustrated in Fig. 4 with mean bias = 0.0035 (95%CI: − 0.072, 0.079), upper LOA = 0.43(95%CI: 0.30, 0.56) and lower LOA =  − 0.42 (95%CI: − 0.29,-0.55). The ICC was 0.87 (*p* < 0.001). High reproducibility of the fibrotic score was demonstrated.

### Correlation

**Fig. 5:**
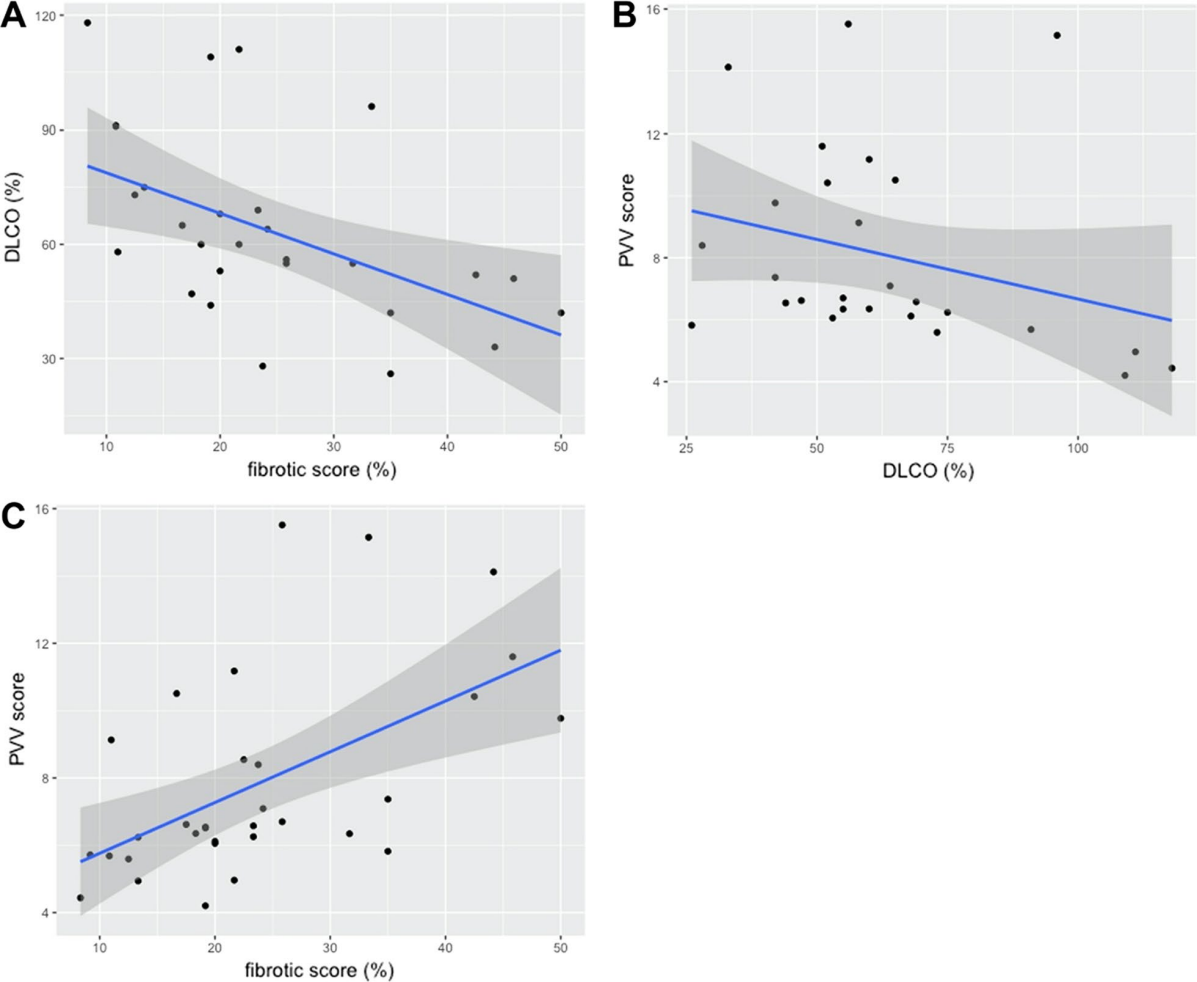
The correlation between the PVV score, fibrotic score and DLCO. **A** The correlation between the fibrotic score and DLCO (r =  − 0.59, *p* = 0.001) **B** the correlation between the PVV score and DLCO (r =  − 0.43, *p* = 0.03) **C** the correlation between the PVV score and fibrotic score (r = 0.59, *p* < 0.001)

The correlation between lung function and CT parameters was demonstrated in the correlation matrix (Table 2). The fibrotic score was correlated with the PVV score (r = 0.59, *p* < 0.001) and DLCO (r =  − 0.59, *p* = 0.001) level. No significant correlation was observed between the fibrotic score and lung volume parameters, including FVC (r =  − 0.20, *p* = 0.3), and CT lung volume (r = 0.09, *p* = 0.62) and PA trunk diameter (r = 0.19, *p* = 0.24). Apart from the fibrotic score, the DLCO levels were also significantly correlated with PVV score (r =  − 0.43, *p* = 0.03). The correlation between the PVV score, fibrotic score and DLCO level is presented in Fig. 5. In addition, DLCO and FVC did not demonstrate a significant parallel trend (r = 0.36, *p* = 0.06), and FVC and CT lung volumes were concordant (r = 0.51, *p* = 0.005). Since the DLCO is composed of KCO and VA. The correlation of PVV score to KCO and VA was further explored. The PVV score did not correlate significantly to KCO (r = -0.30, *p* = 0.13). Instead, the VA correlated to PVV score significantly (r = -0.41, *p* = 0.03). The PA trunk diameter was significantly positively correlated with the PVV score (r = 0.47, *p* = 0.006) but not with FVC (r =  − 0.11, *p* = 0.58) and DLCO (r = 0.22, *p* = 0.28) levels.

**Table 2:** Correlation matrix

	FVC (%)	FEV1 (%)	DLCO (%)	CT-LV	FS	PVV score	PA
FVC (%)	1						
FEV1 (%)	0.88 (< 0.001)	1					
DLCO (%)	0.36 (0.06)	0.22 (0.28)	1				
CT-LV	0.51 (0.005)	0.30 (0.12)	0.32 (0.10)	1			
FS	− 0.20 (0.30)	− 0.06 (0.76)	− 0.59 (0.001)	− 0.09 (0.62)	1		
PVV score	− 0.30 (0.11)	− 0.16 (0.40)	− 0.43 (0.03)	− 0.66 (< 0.001)	0.59 (< 0.001)	1	
PA	− 0.11 (0.58)	0.002 (0.99)	− 0.29 (0.14)	− 0.25 (0.17)	0.19 (0.24)	0.47 (0.006)	1

Data were expressed as r (*p*-value)

*FS* fibrotic score, *PVV* pulmonary vessel volume, *CT-LV* CT measured lung volume, *PA* Pulmonary artery trunk diameter

## Discussion

This study was conducted to evaluate the relationship between the extent of fibrosis, pulmonary vasculature and lung function in patients with IPF. Our current findings may be twofold. The fibrotic score estimated through CT was significantly correlated with DLCO and PVV scores, but not with FVC levels. The DLCO was significantly correlated with the PVV score but did not reveal a parallel trend with the FVC.

In previous studies, the extent of fibrosis in MDCT was found to be correlated with DLCO and FVC levels [12, 16]. This discrepancy with our results may be attributable to two reasons. First, the study population of our study had preserved their FVC despite the fibrosis evident on the CT images; this may have led to an insignificant association between FVC and the extent of lung fibrosis. In contrast, the DLCO was found to have remarkably reduced in our study. It has been observed that the lung volume and DLCO levels may not always change in parallel in IPF [17, 18]. A proportion of patients with IPF had preserved their FVC but had remarkably lower DLCO levels. Moreover, DLCO levels can decline earlier and more seriously than FVC in patients with IPF [18]. Traditionally, a decline in FVC was used as the primary end-point in previous clinical trials [19–21]. Recent studies have reported that FVC alone cannot sufficiently define the severity of IPF and DLCO could shed light on another important physiological defect in IPF, which might be associated with the early diagnosis of fibrosis [22]. Second, the fibrotic score indicates the extent of pulmonary fibrosis but does not distinguish different fibrotic patterns. In Fraser et al. and Wells et al.’s studies, the extent of fibrosis more significantly correlated with DLCO than with FVC levels [12, 23]. These findings might reflect the fact that the influence of fibrosis on lung function is complex in IPF. The extent of fibrosis may have different impacts on FVC based on disease severity. Instead, it affected the DLCO more profoundly.

Jacob et al. reported that the PVV score was significantly correlated with the fibrotic score and DLCO using both CALIPER software and visual fibrotic score [16], which is concordant with our results. However, there were some distinct features of our study. Firstly, in our study, all subjects belonged to the same race (Taiwanese), and patients with moderate and severe emphysema were excluded. Furthermore, the diagnosis of all patients included in this study was confirmed by in the MDD. Through these processes, the well-known high heterogeneity of patients with IPF can be reduced. Finally, we did not find a correlation between PVV scores and FVC as the study of Jacob et al. It may be explained by the fact that PVV score reflected the extent of pulmonary fibrosis (fibrotic score) and the fibrotic score correlated with DLCO but not FVC in this study.

The cause of the correlation between the PVV and fibrotic scores remains unclear. Jacob et al. suggested that the high intrathoracic pressure generated by non-compliant fibrotic lungs dilates the pulmonary vasculature, which might lead to a high PVV score in the fibrotic lung. Contrary to Jacob et al.’s studies, neither PVV nor fibrotic scores were correlated with FVC in our study. FVC negatively reflect lung compliance in IPF [24]. Despite the lack of relevance to FVC, the correlation of the PVV score with fibrotic score remains robust in our study. To further explore the underline mechanism of the negative correlation between PVV score and DLCO, the correlation between PVV score, KCO and VA was performed. It was found that PVV score correlated significantly to VA (r = -0.41, *p* = 0.03) but not KCO (r = -0.3, *p* = 0.13). The negative correlation between PVV score and DLCO in IPF may be explained by the fact that the VA but not KCO was significantly decreased. The low VA may cause expanded PVV score (PVV score is the result of vascular volume divided by total lung volume) and reduced DLCO (DLCO is composed of VA and KCO). However, the effects of fibrosis on the lung vessel volume may be complex. It may be related to pulmonary hypertension, architectural distortion due to fibrosis or vasculature concurrent with fibrosis. Further studies are warranted to illustrate the pathophysiology responsible for the increased PVV score in IPF.

The PA trunk diameter was believed to indirectly reflect pulmonary arterial pressure (PAP) [25]. In our study, we found that the PA trunk diameter was significantly correlated with the PVV score (r =  − 0.47, *p* = 0.006) but not with the fibrotic score (r = 0.19, *p* = 0.24). These findings were concordant with those of previous studies. Jacob et al.’s study also indicated the correlation of the PVV score with the right ventricular systolic pressure measured using echocardiography [16]. Fisher et al.’s study conducted using the right heart catheter to measure PAP also demonstrated that the extent of fibrosis was not associated with PAP in ILD [26]. Although the pulmonary vasculature changes in IPF are still not fully illustrated, a recent study indicated that the pathogenetic process of pulmonary fibrosis may also be responsible for vasculature changes [27]. Another study by Jacob et al. reported that a higher PVV score was related to increased fibrosis and higher mortality [8]. The PVV may be an important radiological marker for interstitial pneumonitis and needs further investigation to verify its significance in IPF.

In our study, FVC levels were found to be significantly correlated with CT lung volume. As the IPF is primarily a disease affecting elderly individuals, the lung function test may be too laborious for some, especially those with cognitive deficiencies [28, 29]. For patients with IPF who have difficulty performing lung function tests, the measurement of CT lung volume may be a possible surrogate to evaluate lung mechanics. As the decline in FVC was used as the physiological marker for disease progression and prognosis prediction, reduced CT lung volume may serve as a quantitative image marker for the same purpose.

This study has some limitations. First, the sample size was small. Most of the included patients did not have reduced FVC. Considering the disease heterogeneity in IPF, our study cohort may only represent a specific proportion of the IPF population (especially those with preserved FVC levels), and the result may not be applicable to all patients with IPF. Second, fibrotic score measurement is a semi-quantitative method that uses the average percentage of fibrosis at six levels on chest CTs to quantify the severity of fibrosis of the entire lung. Despite its high reproducibility among well-trained assessors (both in our study and previous studies), there is a limitation in using a few CT slices to represent the severity of fibrosis of the entire lung. Third, there is still potential to capture the misclassified reticular pattern score among the PVV in patients with extensive fibrosis. Fourth, this study did not obtain information on cardiac echography or right heart catheterisation. Therefore, an association between PA pressure, PA trunk diameter and pulmonary vascularity could not be further illustrated. Finally, because the lung function parameter is expressed by the percentile of expected values while considering the age, sex, body height and weight, a multivariable analysis of the basic characteristics was not performed. Furthermore, CT parameters including fibrotic score, PVV score, PA diameter and CT lung volume were correlated with each other. Considering the problems of collinearity, we could not perform a multivariate analysis of the CT parameters.

## Conclusion

Our study demonstrates the extent of fibrosis and the association of PVV with DLCO but not with FVC levels among patients with IPF. The pulmonary trunk diameter indicated that the PAP was associated with PVV scores. This provides evidence of the structural (image findings of HRCT) and functional association (lung function test) in IPF.

## Data Availability

The datasets used and/or analysed during the current study are available from the corresponding author on reasonable request.

## Abbreviations

IPF: Idiopathic pulmonary fibrosis
HRCT: High-resolution computed tomography
PA: Pulmonary artery trunk
PVV: Pulmonary vascular volume
FVC: Forced vital capacity
DLCO: Diffusing capacity for carbon monoxide
MDD: Multidisciplinary discussion
CTD-ILD: Connective tissue disease-related interstitial lung disease
LAM: Lymphangioleiomyomatosis
LIP: Lymphocytic interstitial pneumonia
CHP: Chronic hypersensitivity pneumonitis
MDCT: Multidetector computed tomography
ICC: Intraclass correlation coefficient
PAP: Pulmonary arterial pressure
